# Supplementary material for: Getting More Out of Biomedical Documents with GATE's Full Lifecycle Open Source Text Analytics
Source: PLoS Comput Biol. 2013 Feb 7;9(2):e1002854. doi: 10.1371/journal.pcbi.1002854 (PMC3567135; doi:10.1371/journal.pcbi.1002854)
Supplement: Dataset S1 — GATE software. Dataset S1 bundled with this paper contains a distribution of GATE (or see http://gate.ac.uk/download/). (TGZ) [file pcbi.1002854.s001.tgz › plos-gate/doc/bibtex.html]

BibTeX to help you create citations if ..


# BibTeX to help you create citations if ..

I used GATE:

```
@Book{Cunningham2011a,
  author = {Hamish Cunningham and Diana Maynard and Kalina Bontcheva and Valentin Tablan and Niraj Aswani and Ian Roberts and
    Genevieve Gorrell and Adam Funk and Angus Roberts and Danica Damljanovic and Thomas Heitz and Mark A. Greenwood and
    Horacio Saggion and Johann Petrak and Yaoyong Li and Wim Peters},
  title = {{Text Processing with GATE (Version 6)}},
  isbn = {978-0956599315},
  year = 2011,
  url = {http://tinyurl.com/gatebook}
}
```

I used ANNIE:

```
@InProceedings{Cunningham2002,
  author = {Hamish Cunningham and Diana Maynard and Kalina Bontcheva and Valentin Tablan},
  title = {{GATE: A Framework and Graphical Development Environment for Robust NLP Tools and Applications}},
  booktitle = {Proceedings of the 40th Anniversary Meeting of the Association for Computational Linguistics (ACL'02)},
  year = 2002
}
```

I used the ontology features in GATE:

```
@Article{Bontcheva2004,
  author = {Kalina Bontcheva and Valentin Tablan and Diana Maynard and Hamish Cunningham},
  title = {{Evolving GATE to Meet New Challenges in Language Engineering}},
  journal = {Natural Language Engineering},
  volume = 10,
  number = {3/4},
  pages = {349--373},
  year = 2004
}
```

I used GATE Mímir:

```
@InCollection{Cunningham2011b,
  author = {Hamish Cunningham and Valentin Tablan and Ian Roberts and Mark A. Greenwood and Niraj Aswani},
  title = {{Information Extraction and Semantic Annotation for Multi-Paradigm Information Management}},
  booktitle = {{Current Challenges in Patent Information Retrieval}},
  publisher = {Springer},
  series = {The Information Retrieval Series},
  volume =   29,
  editor = {Mihai Lupu and Katja Mayer and John Tait and Anthony J. Trippe},
  year = 2011
}
```

I used GATE for Information Retrieval

```
@InProceedings{Cunningham2010,
  author = {Hamish Cunningham and Allan Hanbury and Stefan R\"{u}ger},
  title = {Scaling up High-Value Retrieval to Medium-Volume Data},
  booktitle = {Advances in Multidisciplinary Retrieval (the 1st Information Retrieval Facility Conference)},
  month = {May},
  series = {Lecture Notes in Computer Science, Volume 6107},
  publisher = {Springer},
  address = {Vienna, Austria},
  editor = {Hamish Cunningham and Allan Hanbury and Stefan R\"{u}ger},
  isbn = {978-3-642-13083-0},
  year = 2010
}
```

I used GATE with multimedia:

```
@InProceedings{Dowman2005,
  author = {Mike Dowman and Valentin Tablan and Hamish Cunningham and Borislav Popov},
  title = {Web-Assisted Annotation, Semantic Indexing and Search of Television and Radio News},
  booktitle = {Proceedings of the 14th International World Wide Web Conference},
  address = {Chiba, Japan},
  url = {http://gate.ac.uk/sale/www05/web-assisted-annotation.pdf},
  year = 2005
}
```

I used the machine learning facilities in GATE:

```
@Article{Li2009,
  author = {Yaoyong Li and Kalina Bontcheva and Hamish Cunningham},
  title = {{Adapting SVM for Data Sparseness and Imbalance: A Case Study on Information Extraction}},
  journal = {Natural Language Engineering},
  volume = 15,
  number = 2,
  pages = {241--271},
  url = {http://journals.cambridge.org/repo_A45LfkBD},
  year = 2009
}
```
